# Impact of microplastics on female reproductive health: insights from animal and human experimental studies: a systematic review

**DOI:** 10.1007/s00404-024-07929-w

**Published:** 2025-01-16

**Authors:** Özen Inam

**Affiliations:** Department of Medical Services and Techniques, Maltepe University, Başıbüyük, Maltepe, Istanbul, Turkey

**Keywords:** Microplastics, Female reproductive health, Fertility, Ovarian function, Embryo development

## Abstract

**Objective:**

This systematic review aims to evaluate the impact of microplastics on female reproductive health by analyzing experimental studies.

**Method:**

A comprehensive search was conducted in PubMed, Web of Science, and Scopus databases to identify experimental studies published between 2021 and 2023. Studies investigating the effects of microplastics on reproductive organs, hormone levels, fertility rates, and offspring development in female subjects were included. The quality of the studies was assessed using the Cochrane risk of bias tool.

**Results:**

A total of 15 studies met the inclusion criteria. The results indicate that exposure to microplastics significantly affects ovarian function, decreases fertility rates, and disrupts hormone levels in female subjects. Several studies also reported negative effects on embryo development and offsprings health. The quality of the studies varied, with some showing a high risk of bias.

**Conclusion:**

The evidence from experimental studies suggests that microplastics have a detrimental effect on female reproductive health. However, the variation in study quality highlights the need for more rigorous research to confirm these results and better understand the underlying mechanisms.

## Introduction

In recent years, during what is known as the Anthropocene epoch, human activities have significantly impacted nature, posing numerous threats to both the environment and humanity Among these, plastic pollution has emerged as a global issue, representing a pervasive threat driven by human actions [1]. As concerns about plastic waste disposal and environmental pollution grow, a less visible but equally serious threat has emerged: microplastics. These particles not only endanger our environment but also pose significant risks to human and animal health [2].

Microplastics originate from various sources. The breakdown of plastic waste, microbeads in cosmetic products, washing of textiles, and industrial activities contribute to the dissemination of these microscopic particles into the environment [2–4]. These particles infiltrate water sources and spread through the food chain, thereby posing potential risks to both humans and animals. Humans can ingest microplastics directly or indirectly, such as through the consumption of marine life, which has absorbed these particles from the food chain.

Research into the effects of microplastics on human health, particularly concerning the respiratory, digestive, and circulatory systems, has revealed alarming results. When these microplastic particles, along with the chemicals they contain, enter the body, they may lead to long-term health problems [5].

The situation is no different for female animals. Marine organisms, in particular, tend to be exposed to microplastics within aquatic ecosystems. This exposure can have detrimental effects on reproductive systems and threatens the sustainability of species. Female animals are especially crucial for the continuation of species and the maintenance of a healthy reproductive system [6, 7].

This paper examines experimental studies that demonstrate the potential effects of microplastics on the reproductive health of female animals and humans. In light of scientific results, we aim to understand the severity of this microscopic threat and contribute to raising awareness within society. Measures taken to reduce the impacts of microplastics on health will not only protect the health of individuals and communities but also contribute to the sustainability of ecosystems.

## Materials and methods

### Literature search parameters

**Fig. 1:**
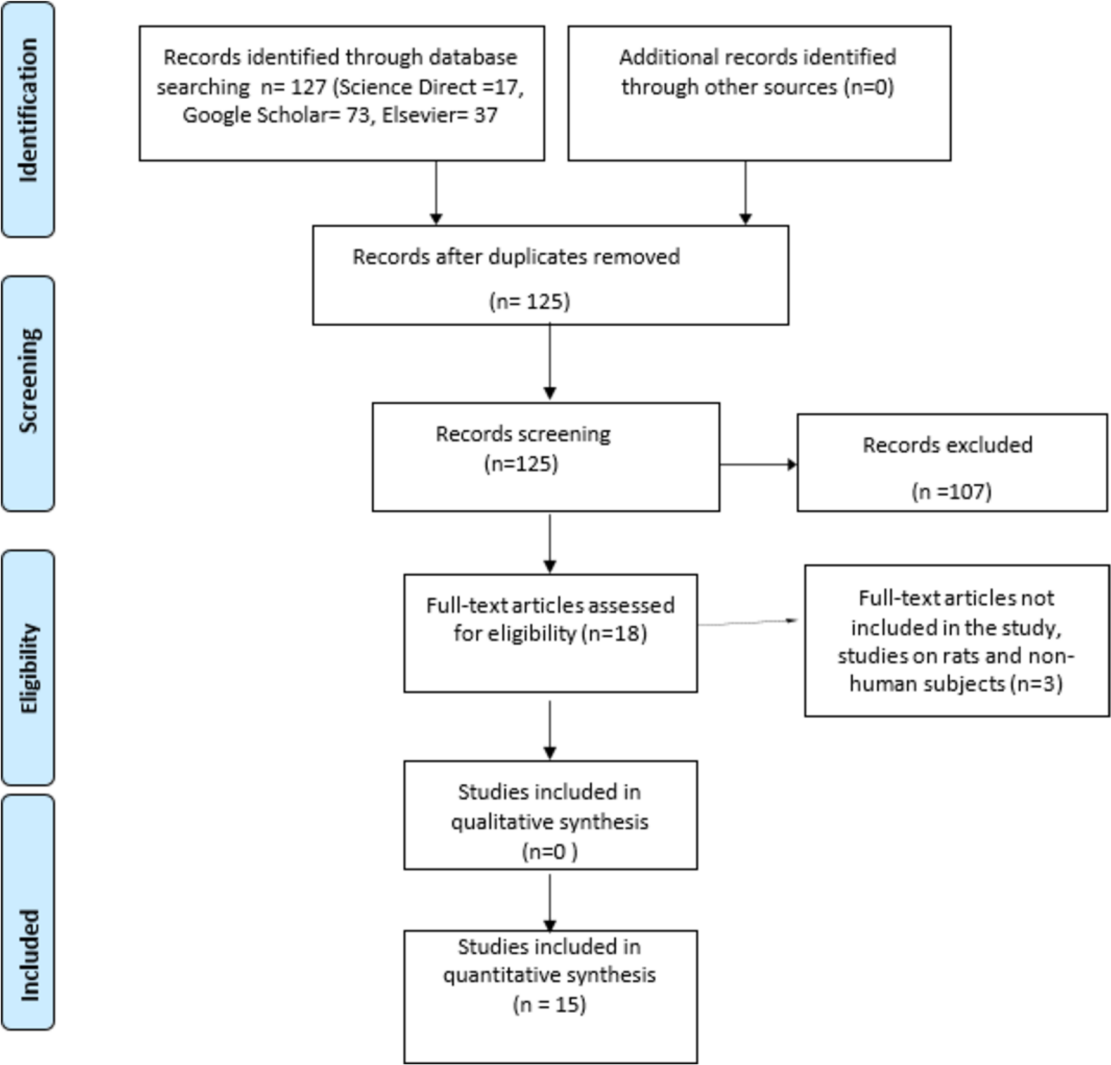
Summary of article inclusion and screening using the PRISMA flowchart (Moher et al., 2009)*. *From: Moher D, Liberati A, Tetzlaff J, Altman DG, The PRISMA Group (2009). *P*referred *R*eporting *I*tems for *S*ystematic Reviews and *M*eta- *A*nalyses: The PRISMA Statement. PLoS Med 6(7): e1000097. doi:10.1371/journal.pmed1000097

The design of this systematic review follows the guidelines of Siddaway et al. (2019). The main literature search was conducted between October and November 2023. The literature searches were carried out using online publication databases: “ScienceDirect, Elsevier, and Google Scholar”. The selection process of the articles was summarized according to the PRISMA approach in Fig. 1.

At the beginning of the study, the aim and scope were clearly defined. The objective of the study was to identify the potential threats that microplastics might pose to the female reproductive system. Therefore, articles containing the keywords “microplastics and female health” were searched, with the condition that they were experimental studies. The initial goal was to access all studies related to the topic. However, due to the large number of studies found and the irrelevance of some to the specific topic, further refinements were made.

The term “female animals” was restricted to *"rats," "mice,"* and *"humans"* because small marine organisms were numerous and diverse, and were not suitable for comparison with humans (e.g., organisms like water flies). Keywords were used in English.

- *Subject:* "Microplastics," "Phthalates," "Bisphenol"
- *Target:* Human ∗ , Rat ∗ , Mouse ∗ , Female reproductive health

The terms in each category ("subject" and "target") were combined using "OR" and then further combined using "AND." The full search string reads as follows: “Microplastics” ∗ OR “Phthalates” ∗ OR “Bisphenol” ∗) AND (Human ∗ OR Mouse ∗ OR Rat ∗ AND Female reproductive health.

### Study screening process

The articles found during the searches were evaluated for inclusion using a two-stage screening process. These stages were "selection of those meeting the inclusion criteria and review and summarization of data from the selected articles."

### Step 1: Study inclusion criteria

The title and abstract of each publication were evaluated for relevance using a set of inclusion criteria:

- Subject: Does the article address the effects of microplastic exposure on female reproductive health in humans, rats, or mice?
- Results: Does the article provide experimental results regarding the interaction between female organisms and microplastics?
- Study type: Is it an experimental study published in a peer-reviewed journal?
- Duplicate articles: Is the same article found under the same title in three different databases?

In summary, the following inclusion criteria were considered in the selection of articles for the study:

- The research examines the impact of microplastics on the female reproductive system/health.
- It is an experimental study.
- The research was published from 2022 onwards.
- The study is accessible in full text and is neither a review nor a meta-analysis.

### Step 2: Data extraction and summarization

Potentially relevant articles were read in full, and information pertinent to this review was extracted from the appropriate articles. When available, details about the study method, target species, and the nature and characteristics of microplastic exposure were collected. The extracted data were organized and evaluated using a data summarization table for clarity. The studies included in the review were summarized according to the data summarization form, under the headings: title, objective, method, results, and conclusion. The detailed list of variables and minimum requirements is provided in Table 1.

**Table 1:** Key Findings and Data from Experimental Studies on Microplastics and the Female Reproductive System

Characteristics	Categories
Sample type and number examined	Rat-mouse / Human tissue-fluid
Organ/system examined	Uterus / Vagina / Ovaries / Oocytes / Mammary gland / Breast milk / Blood / Urine
Country, Year, Author Information	Country/City, Year, Authors
Method*	
Polymer/mikroplastik görüntüleme yöntemi	Raman spectroscopy; Electron microscopy
Polymer/microplastic type	Bisphenol, Polystyrene microplastics (PA: poliamid; PU: poliüretan; PE: polietilen; PET: polietilen tereftalat…)
Contamination method	Food / Water / Air / None
Main Results	Relationship of microplastics with reproductive system tissues, organs, and fluids

^*^ Emphasizes the minimum information requirements that studies must include

The health outcomes identified in the results were also summarized. Methodological differences and similarities between the studies were then discussed.

## Results

Searches conducted using the main search terms in three databases resulted in a total of 127 articles. Two articles were excluded because they were duplicates. After screening titles, abstracts, and methods, 107 articles were excluded. Of these, 20 articles were excluded because they were not related to the reproductive system, 18 because they focused on the male reproductive system, 19 because they were related to chemicals other than microplastics, 4 because they were meta-analyses, and 22 because they were reviews. Additionally, 24 articles were excluded because they were conducted on non-rodent species (e.g., zebrafish, worms). Furthermore, the full texts of 3 articles were not accessible. As a result, 15 research articles were comprehensively included in the review (Aghaei et al., 2022; Amereh et al., 2022; Di Bella et al., 2023; Grechi et al., 2023; Haddadi et al., 2022; Hou et al., 2021; Liu et al., 2022; Liu et al., 2023; Zhang et al., 2023; Raghavan et al., 2023; Ragusa et al., 2022; Segovia-Mendoza et al., 2022; Tiao et al., 2023; Yang et al., 2022; Zaheer et al., 2022).

The sampling methods, research methods, findings, and results of the studies addressed in our work are presented in more detail in Table 2.

**Table 2:** Summary of Methods and Results of Experimental Studies Investigating the Effects of Microplastics on the Female Reproductive System

Author & Year	Sample type & number	Microplastic types & exposure	Imaging method	Reproductive system tissue-organ-fluids relation			Results
				*Embriyo \Fetus*	*Placenta / Amniotic Fluid / Meconium / Breast Milk*	Reproductive Organs	
[20] Mexico	*Human serum; Women in n* = *102 (37 without disease, 65 with breast cancer)*	Diethylhexyl phthalate (DEHP), butylbenzyl phthalate (BBP), dibutyl phthalate (DBP), diethyl phthalate (DEP), bisphenol A (BPA) levels, and bisphenol S (BPS)	Serum concentration measured by gas chromatography-mass spectrometry	x	x	**In women with breast cancer,** different increases in serum phthalate concentrations were observed compared to women without the disease. Lower BBP levels were found in survivors compared to active patients. p < 0.05	Supports the hypothesis that there is a positive relationship between exposure to phthalates and the incidence of breast cancer
[7]	*Human placenta from* *10 women who had given birth*	Not exposed. The presence of substances attributed to plastics was visually identified in the villi and cellular structure of the placenta	Scanning Electron Microscope (SEM) Transmission Electron Microscope (TEM)	x	Microplastics (MPs) were first detected in the **human placenta** using electron microscopy The presence of MPs was associated with changes in certain cell organelles within the placental tissue. Unreported changes in the morphology of the endoplasmic reticulum and mitochondria were found in healthy term pregnancies	x	Microplastics in the human placenta may contribute to the activation of pathological features such as oxidative stress, apoptosis, and inflammation Microplastics in the human placenta could have long-term effects on human health
[17] China	*Human maternal and infant materials* *n* = *12 (breast milk and meconium)*	PA: Polyamide PU: Polyurethane PE: Polyethylene PE:Polyethylene Terephthalate PVC: Polyvinyl Chloride PPPolypropylenePTFE:Polytetrafluoroethylene PC:Polycarbonat	8700 laser infrared imaging spectrometer (LDIR), quantum cascade laser (QCL)	x	16 types of MPs were detected in all samples x PA and PU accounted for more than 65% of the total amount of MP particles. The most common MPs in **placenta, meconium, and infant feces** were PA, constituting 50.09%, 60.22%, and 49.67% respectively. Dominant MPs in breast milk and infant formula were PU, with 53.18% and 49.33% respectively. More than 74% of the MP particles found in all samples were 20–50 μm in size		Various MPs were found in the placenta, meconium, infant feces, and breast milk, with PA and PU being the most dominant. Water intake and the use of cleaning agents or toothpaste may expose pregnant women, while breast milk, bottle use, and plastic toys may expose infants
[8] Canada	*Pregnant Mice 40 healthy female mice (7–16 weeks old) were used for mating* *3 different concentrations and 1 control group*	Polystyrene (PS-MP), Polystyrene Nanoplastics (PS-NP') Mice were exposed to 5 μm PS-MPs and 50 nm polystyrene PS-NPs in filtered drinking water	Placental metabolite profiles were determined using high-resolution magic angle spinning magnetic resonance spectroscopy	**In fetuse:** exposed to MPs and NPs, a significant dose-dependent reduction in fetal weights was found (p < 0.0001)	Maternal exposure to PS-MPs and PS-NPs x affected the structure of the **placenta** and resulted in a shortening of the umbilical cord length. With increasing MP concentration, there was a relative decrease in lysine (p = 0.003) and glucose (p < 0.0001) in the placenta		Maternal exposure to microplastics has led to significant changes in placental metabolism. This study highlights the potential effects of microplastic exposure on pregnancy outcomes
[15]	*Wistar female rats, with 21 estrous cycles and weighing 196* ± *11 g, were divided into two groups: one exposed to MPs (n* = *14) and a control group with distilled water (n* = *7)*	Polystyrene microplastics (PS-MPs): Fluorescent PS-MPs were given to 7 animals, and 7 animals received PS-MPs via oral gavage (0.1 mg/day, 1.5 × 10^6 particles/day)	Sections were imaged using an epifluorescence microscope	x	x	FPS-MPs were observed in the **ovaries and follicles**. Compared to the controls, a significant reduction in the relative ovarian weight was recorded in the group exposed to PS-MPs (p < 0.05). Exposure to PS-MPs was found to significantly decrease the duration of the fourth estrous cycle compared to the control group (p < 0.01)	The study provides initial evidence that exposure to PS-MPs leads to abnormal estrous cycles and defective folliculogenesis in rats. One significant finding is the detection of PS-MPs in various ovarian sections and their adverse effects on the expression of α-tubulin and DAAM-1 proteins
[12] Czech Republic	*Human amniotic fluid and placenta: 10 cases of early membrane rupture (20 samples of amniotic fluid and placenta)*	44 different types of microplastics were found	Analysis of the particles separated from the samples was performed using Fourier-transform infrared spectroscopy with an integrated microscope on a Nicolet iN10 device	x	**Placenta and amniotic fluid**: All amniotic fluid samples contained 16 particles of microplastics or polymer additives. Among the polymer components, Chlorinated Polyethylene (CPE) and Calcium Zinc PVC stabilizer were prominent	x	Microplastics or additives were found in amniotic fluid, placenta, or both in 9 out of 10 patients. In 7 of these 10 patients, a lower number of particles were observed in the amniotic fluid compared to the placenta, suggesting that the placenta may act as a partial barrier against the entry of microplastics into the amniotic fluid and fetus
[11]	*Human and bovine follicular fluid: 7 follicular fluid samples obtained from human specimens*	Polystyrene MP (PS-MS)	Raman Spectroscopy: Confocal Raman (alpha300 R, WITec, Germany) with a 532 nm laser	x	x	**Human Follicular Fluid:** A total of 47 different MP polymers were identified, with rubber being the most common in humans. MP polymers accounted for 0.8% to 23.5% of the total number of particles identified. Other identified plastic-related particles included pigments (0% to 3.6%), plasticizers (2.0% to 50.9%), and coatings, solvents, and fillers (2.8% to 33.3%)	The amount of MP microplastics found in human follicular fluid was measured, and their experimental effects on bovine oocytes were investigated. It was found that the levels of MPs detected in human follicular fluid were sufficient to adversely affect oocyte maturation
[10]	*43 human placentas*	The majority of the MPs consisted of PE (polyethylene) and PS (polystyrene)	Digital microscopy and Raman microspectroscopy	**Fetal Effects:** Exposure to MPs showed inverse relationships with birth outcomes, including birth weight (r = -0.82, p < 0.001), length (r = -0.56, p < 0.001), and head circumference (r = -0.50, p = 0.001)	**Placental Examination:** x MPs were found in all pregnancies with intrauterine growth restriction (13 out of 13), with an average abundance ranging from 2 to 38 particles per placenta. However, in normal pregnancies, MPs were detected at levels below the detection threshold in 3 out of 30 samples		In placentas with IUGR (intrauterine growth restriction), the abundance of MPs was found to be higher compared to normal pregnancies. An increase in MPs was associated with reductions in birth weight, length, head circumference, and Apgar scores. These results suggest that microplastics in the placenta may adversely affect fetal development
[21]	*Pregnant mice and their fetuses (experimental group of 5 mice)*	Polystyrene (PS) particles: Green/red fluorescent microspheres of 100 nm size (10 mg/mL solution) were administered via intragastric gavage	The size and shape of PS particles were determined using a scanning electron microscope (SEM; JSM-6390LV)	Maternal exposure to PS particles led to anxiety-like behaviors in the **babie**s and a reduction in γ-aminobutyric acid (GABA) in the prefrontal cortex and amygdala at week 8. PS nanoparticles were found to accumulate in the thoracic and abdominal organs of the **fetuses.** Surprisingly, higher fluorescence intensity of PS nanoparticles was observed in the fetal brain. The effects were further enhanced when fetuses were exposed to a mixture of NPs and MPs		x x	PS nanoparticles penetrated the fetal thalamus through the blood-placenta barrier. PS particles during pregnancy led to anxiety-like behaviors in the offsprings and a decrease in GABA levels. It is believed that MPs damage the placental barrier, facilitating the easier entry of PS nanoparticles into the fetal brain
[16]	*32 Wistar rats*	Exposed to different concentrations of 0.5 µm PS MPs dispersed in deionized water (drinking water) (0.15 mg/kg/day and 1.5 mg/kg/day)	The shape of the particles was observed using a scanning electron microscope (SEM, JEOL/EO)	x	x	**Ovarian** images showed that PS MPs were located in the cytoplasm of granulosa cells. PS microplastics were found to induce pyroptosis and apoptosis in granulosa cells. Compared to the control group, the MP groups caused a significant decrease in AMH levels and resulted in thinner granulosa layers in some secondary follicles	Polystyrene microplastics can enter the rat ovary and be taken up by granulosa cells. The exposure to microplastics has been suggested to have adverse effects on the ovary and may represent a potential risk factor for female infertility
[22]	*55 control and 55 PE group mother rats* *10 control and 10 PE baby rats*	Polyethylene (PE; IUPAC name: polyethene or polymethylene Administered orally with PE using 100 μL PBS	**Brain samples** were examined using a laser scanning confocal microscope with a fluorescent filter (LSM-710, Zeiss) and Fourier-transform infrared spectroscopy (FT-IR)	Even after 1 week of oral administration of 100 ppm/100 µL PE, it was found that PE accumulated in the brain, as observed using confocal microscopy and SEM	The microbiome of the PE group x **babies** was found to be different from that of the control group. Dilution curves showed that richness was 84.88 for the control and 49.95 for PE (n = 5, *P < 0.005). Molecular evidence of OSB-like features was observed as metabolite changes identified by magnetic resonance in the prefrontal cortex and hippocampus regions. Heat maps indicated changes in RNA gene expression in the prefrontal cortex and hippocampus after exposure to PE		Exposure to PE during pregnancy led to autism spectrum disorder (ASD)-like features in offsprings, including impaired social interactions, repetitive behaviors, and disruptions in brain metabolite levels and gene expression. It also caused changes in the gut microbiome, resulting in ASD-like characteristics in the mice
[13]	*Pregnant Sprague–Dawley rats (8 pregnant rats)*	Exposure to a high-fat diet (HFD) and/or microplastics in the diet	The Leica DMI-3000 microscope was used	The body weight of the **pups** x in all experimental groups increased compared to the control group after prenatal exposure to microplastics. It was found that the ileum length of the pups in the MP experimental group was shorter compared to the control group. Additionally, oxidative stress and apoptosis were increased in the liver of pups exposed to microplastics prenatally		It was found that the liver weight of the **mother rats** increased compared to the control group due to both high-fat diet and microplastic exposure (p < 0.05)	Prenatal exposure to microplastics along with a high-fat diet (HFD) led to additional increases in liver lipid accumulation in the offsprings. Prenatal exposure to HFD and high concentrations of microplastics resulted in a reduction in ileum villus length, and an increase in liver apoptosis and inflammation compared to the control
[14]	*40 female rats (C57BL/6 J)*	PE-MPs were administered orally to the parent rats via gavage for 30 days	The investigation was conducted using transmission electron microscopy (TEM) and light microscopy. Tissue pathology was assessed through histopathological analysis	Maternal exposure to high doses of PE-MPs resulted in adverse effects on **embryonic** development, with a reduction in birth and postnatal body weight in the **babies**	x	PE-MPs increased the levels of reactive oxygen species (ROS) and DNA damage in **oocytes,** impaired mitochondrial function, and induced apoptosis. This led to a decrease in oocyte quality and a reduction in fertility	Microplastics have been shown to exert severe toxic effects on female reproductive health, with these effects occurring through mechanisms such as oxidative stress and mitochondrial dysfunction. The number of live births and the body weight of the offsprings were affected
[19] Sicily	*Human Blood: 75 Sicilian women (reproductive age, premenopausal, and menopausal periods)*	phthalates (PAEs), non-phthalate plasticizers (NPPs), and bisphenols (BPs)	Bisphenols were extracted from blood samples using acetonitrile and water, while plasticizers were extracted using distilled water, sodium chloride, and hexane	x	Common plasticizers (PAEs and NPPs) x and bisphenols (BPs) were detected in **women's blood**. DEHP and DIBP were the most frequently found plasticizers across all age groups, while BPA and BPS were the most common bisphenols. The levels of these chemicals were higher in younger women (20–44 years) compared to menopausal women (51–60 years)		DEHP and DIBP are the most commonly found plasticizers across all age groups. BPA and BPS are the main bisphenols detected in all blood samples. Levels of plasticizers and bisphenols are higher in younger women compared to menopausal women
[18] China	*18 pregnant women and their infants were studied*	Sixteen types of microplastics, including various polystyrene and polyethylene varieties, were detected in the placenta and meconium	Agilent 8700 laser infrared imaging spectrometer (LDIR) was used for the identification of MPs	x	Sixteen different types of MPs were x detected in **placenta and meconium** samples. Polyamide (PA) and polyurethane (PU) were the most common types of MPs. 76.46% of the MPs in the samples were sized 20–50 μm. The size of MP particles was related to the meconium microbiota. PA and PU were found to be inversely related to the placenta microbiota		MPs are widespread in placenta and meconium samples, indicating extensive exposure for pregnant women and infants. There may be a connection between high MP concentrations and microbiota types. Significant relationships have been found between MP particle sizes and the meconium microbiota

### Summary of original articles

Summary of original articles the fundamental methods and results of the reviewed studies are summarized as follows:

- 1. Maternal exposure to polystyrene microplastics alters placental metabolism in mice (Aghaei et al., 2022):
- This research study on mice suggested that exposure to polystyrene microplastics led to changes in placental metabolism, which could affect fetal development. Methods included exposing mice to polystyrene microplastics and analyzing the metabolic profile of the placenta.
- 2. Exposure to microplastics leads to defective ovarian function and changes in cytoskeleton protein expression in rats (Haddadi et al., 2022):
- This study revealed that exposure to microplastics impaired ovarian function and altered cytoskeleton protein expression in rats. The method involved exposing rats to microplastics, followed by an evaluation of ovarian function and cytoskeleton protein expression.
- 3. Detection of various microplastics in placentas, meconium, infant feces, breastmilk, and infant formula: A pilot prospective study (Liu et al., 2023):
- This pilot study demonstrated the detection of various microplastics in placentas, meconium, infant feces, breastmilk, and infant formula. Various biological samples were examined using special analytical methods to detect microplastics. Water intake, use of cleansing agents, or toothpaste could have been potential sources of exposure for pregnant women.
- 4. Polystyrene micro- and nano-particle coexposure injures fetal thalamus by inducing ROS-mediated cell apoptosis (Yang et al., 2022):
- This study showed that exposure to polystyrene micro- and nano-particles in pregnant mice induced apoptosis in the fetal thalamus through ROS. Pregnant mice were exposed to micro- and nano-particles, and PS nanoparticles infiltrated the fetal thalamus through the blood-placenta barrier. Exposure during pregnancy led to anxiety-like behaviors and decreased GABA levels in the offsprings.
- 5. The Association Between Microplastics and Microbiota in Placentas and Meconium: The First Evidence in Humans (Liu et al., 2022):
  This study presented the first evidence of the association between microplastics and microbiota in placentas and meconium, involving 18 mothers and infants. The placentas and meconium were examined using special methods for microplastic detection and microbiota analysis, confirming the presence of MPs.
- 6. Screening of phthalate and non-phthalate plasticizers and bisphenols in Sicilian women’s blood (Di Bella et al., 2023):
- The study identified the profiles of various plastic additives (phthalates, non-phthalate plasticizers, and bisphenols) in the blood of Sicilian women and found a relationship between these chemical levels and age. Younger women were exposed to higher levels of these substances compared to older women. Blood samples were analyzed using various separation methods and evaluated for plasticizers and bisphenols.
- 7. Reproductive toxicity of microplastics in female mice and their offspring from induction of oxidative stress (Zhang et al., 2023):
- This study investigated the effects of polyethylene microplastics (PE-MPs) on female reproductive health. Oral exposure to MPs for 30 days significantly reduced oocyte maturation and fertilization rates, leading to reproductive toxicity. The oxidative stress, mitochondrial dysfunction, and DNA damage caused by MPs compromised oocyte quality and negatively impacted embryo development. Additionally, maternal exposure to MPs resulted in impaired growth and fertility parameters in offsprings.
- 8. Pre/post-natal exposure to microplastic as a potential risk factor for autism spectrum disorder (Zaheer et al., 2022):
- This study explored whether prenatal and postnatal exposure to microplastics posed a risk for autism spectrum disorder in offsprings. It involved examining the accumulation of polyethylene (PE) in the brains of mice and assessing behavioral effects using different life stage models, such as prenatal, post-weaning, adolescent, and adult models. Changes in brain tissues and ASD-like symptoms were observed.
- 9. Polystyrene microplastics lead to pyroptosis and apoptosis of ovarian granulosa cells via NLRP3/Caspase-1 signaling pathway in rats (Hou et al., 2021):
- This study investigated the effects of polystyrene microplastics (PS-MPs) on ovarian function in rats, exposing healthy female rats to MPs for 90 days. Electron microscopy and various staining methods were used, suggesting that exposure to microplastics had negative effects on the ovary, potentially serving as a risk factor for female infertility.
- 10. Placental plastics in young women from the general population correlate with reduced fetal growth in IUGR pregnancies (Amereh et al., 2022):
- This study investigated the presence of microplastics (MPs) in the placentas of pregnant women and examined their impact on newborn growth outcomes. Placentas from 43 women were analyzed using digital microscopy and Raman microspectroscopy, result that MPs, primarily polyethylene (PE) and polystyrene (PS), were more abundant in IUGR placentas than in normal pregnancies.
- 11. Microplastics are present in women’s and cows’ follicular fluid, and polystyrene microplastics compromise bovine oocyte function in vitro (Grechi et al., 2023):
- This study investigated the presence of microplastics in human and bovine ovarian fluids and their potential impacts on reproductive health. The study utilized an optimized protocol for isolating plastics from biological samples and found that polystyrene microplastics in these fluids could adversely affect oocyte maturation.
- 12. Microplastics and additives in patients with preterm birth: The first evidence of their presence in both human amniotic fluid and placenta (Halfar et al., 2023):
  This study examined the presence of microplastics in the amniotic fluid and placenta of pregnant women. Analyses of 20 samples from 10 women revealed the presence of microplastics and polymer additives in both amniotic fluid and placenta.
- 13. Deeply in Plasticenta: Presence of Microplastics in the Intracellular Compartment of Human Placentas (Ragusa et al., 2022):
- The study directly detected the presence and localization of microplastics in human placentas (n = 10), f result that these particles caused damage to cellular organelles and may trigger pathological features by disrupting metabolic processes.
- 14. Prenatal High-Fat Diet Combined with Microplastic Exposure Induces Liver Injury via Oxidative Stress in Male Pups (Tiao et al., 2023):
- A study by Tiao et al. (2023) investigated the combined effects of prenatal microplastic exposure and a high-fat diet (HFD) on liver health in male offspring. Results showed that this combination significantly increased liver steatosis, apoptosis, and inflammation. Higher microplastic concentrations further exacerbated liver lipid accumulation, oxidative stress, and cell death.
- 15. Association of Serum Levels of Plasticizers Compounds, Phthalates, and Bisphenols in Patients and Survivors of Breast Cancer: A Real Connection? (Mendoza et al., 2022):
- This study measured serum levels of phthalates and bisphenols in women from two cities in Mexico to examine their relationship with breast cancer. Concentrations of these chemicals were compared among healthy women, breast cancer patients, and breast cancer survivors. The results showed a positive association between serum levels of phthalates and bisphenols and the incidence of breast cancer.

### Common results from experimental studies

Our research found that the results from the 15 studies analyzed shared common findings under 4 main categories. The key methods and results of the reviewed studies were summarized as follows.

- 1. Effects on placenta and fetal development:
- In 7 out of the 15 studies reviewed, the harmful effects of microplastics on placental and fetal health were assessed. These effects were as follows:
- 2. Two studies found that microplastic exposure altered placental metabolism in mice [8, 9].
- 3. Plastics found in the placenta of young women were suggested to be associated with reduced fetal growth in IUGR pregnancies [10].
- 4. One study examined the presence and effects of microplastics in human and bovine ovarian fluids. It found that polystyrene microplastics were present at levels sufficient to adversely affect oocyte maturation [11].
- 5. The presence of microplastics and polymer additives in amniotic fluid and placenta was revealed [12].
- 6. Plastics were found in both recorded and extracellular compartments of placental tissue, associated with endoplasmic reticulum and mitochondrial injury, and also causing modifications to microvilli [7].
- 7. Exposure to microplastics and a high-fat diet (HFD) during pregnancy was found to increase liver fat accumulation, apoptosis, and inflammation in the fetus [13].
- 8. Polyethylene microplastics were found to impair placental functions and negatively affect fetal health [14].
- 9. Ovarian function and reproductive health:
- Two of the studies reviewed were conducted on rats and revealed the ovarian effects of microplastics on the placenta. These effects are as follows:
- 10. Microplastic exposure was found to impair ovarian function and cause changes in cytoskeleton protein expression in rats [15].
- 11. Polystyrene microplastics were suggested to trigger pyroptosis and apoptosis in ovarian granulosa cells in rats [16].
- 12. The effects of polyethylene microplastics on oocytes and embryos negatively impacted oocyte quality and embryo development, leading to decreased fertility and lower rates of healthy pregnancies [14].
- 13. Detection of microplastics in human tissues and biological samples:
- Five of the studies reviewed focused on the detection of microplastics in human tissues or biological samples.
- 14. Various microplastics were detected in human placenta, meconium, infant feces, breast milk, and infant formula [17].
- 15. The presence of microplastics in the intracellular compartments of human placentas was demonstrated [7].
- 16. This study provides the first evidence of an association between microplastics and the microbiota in placentas and meconium, based on the detection of 16 microplastic types and their correlation with microbiota compositions [18].
- 17. The presence of plasticizers and microplastics was detected in all blood samples from women. Young women had higher levels of plasticizers and bisphenol compared to women in the menopausal period [19].
- 18. Detectable levels of phthalates and bisphenol were found in serum. Especially children and young adults were observed to have higher levels of MP [20].
- 19. Effects on cells/tissues due to oxidative stress:
- In 5 of the reviewed studies, microplastics were found to increase oxidative stress on the immune system and cause damage to cells/tissues.
- 20. Exposure to polystyrene micro- and nanoparticles was found to cause cell apoptosis in the fetal thalamus through ROS [21].
- 21. Polystyrene microplastics were found to trigger pyroptosis and apoptosis in ovarian granulosa cells in rats [16].
- 22. Oxidative stress induced by polyethylene microplastics significantly contributed to reduced oocyte quality and impaired reproductive outcomes [14].
- 23. Exposure led to disruption in brain metabolites and gene expression in young mice, and it also led to changes in gut microbiota, which detected autism spectrum disorder-like features in mice [22].
- 24. The relationship between microplastic exposure and the development of breast cancer was examined, and it was suggested that this exposure could potentially increase the risk of breast cancer [20].

## Discussion

With the increasing use of plastics in modern life, their harmful effects on both nature and human health have become a significant topic of discussion. Plastics are in constant contact with humans and the environment through various means, including textiles, food packaging, healthcare tools, and cosmetic products [23, 24]. Numerous studies have investigated the potential harms of these widely used plastics on human health [6, 25, 26]. However, the effects of microplastics on women’s health hold particular importance. Women play a crucial role in the continuity of generations and significantly influence the health of embryos and fetuses through their roles in fertility and breastfeeding. In this context, this systematic review evaluates experimental studies that have demonstrated the harmful effects of microplastics on female reproductive health.

The results of this systematic review clearly demonstrate the potential harms of microplastics on female reproductive health. In particular, microplastic exposure has been shown to have significant negative effects on ovarian functions, fertility, hormone levels, and embryo development. These results are consistent with the existing literature and are supported by other studies investigating the effects of microplastics on hormonal and reproductive systems [6, 27].

The experimental studies reviewed converge on four common outcomes. The first is the "effects on placental and fetal development." Disruptions in placental tissue, reduced fetal growth, and adverse effects on oocyte maturation are the identified results. Two different review studies have also emphasized the harms of microplastics in this regard [28, 29]. Another experimental study conducted in 2019 found that microplastics caused damage to uterine arteries, increased miscarriage rates, and led to placental disruptions [30]. The second effect found in the reviewed studies is on "ovarian function and reproductive health" [15, 16]. An experimental study conducted on zebrafish in the literature also found that microplastic exposure induced apoptosis in ovaries [31]. Another experimental study conducted in 2021 found that microplastics caused cellular damage and ovarian fibrosis in ovaries [32]. As seen in the studies reviewed in our research, ovarian dysfunction or apoptosis is a significant issue that will directly affect reproductive health.

The human-related studies we reviewed generally focused on the detection of microplastics in tissues or biological samples. In studies conducted on women’s reproductive health, microplastics were detected in the placenta, breast milk, meconium, and blood samples [7, 18, 19]. Qin’s study, conducted in 2024 and with data yet to be fully published, found a wide variety of microplastic types in the endometrial tissue of 22 women [33]. Preliminary data from a study published after our review on October 15, 2024, also reported the detection of microplastics in the blood and cancerous/paracancerous tissues of women with cervical cancer [34]. Beyond the female reproductive system, microplastics have been detected in human tissues such as lung tissue, cirrhotic liver tissue, pericardial, epicardial, myocardial, venous blood, and colectomy samples during or after certain surgeries [35–39].

The reviewed studies suggest that oxidative stress-induced immune system disruption underlies the effects of microplastics on tissues. Review and research articles in the literature also point to microplastics triggering oxidative stress and inflammation in cells, impairing immune functions, and causing neurotoxicity [1, 40–42].

However, methodological differences in the studies, such as the animal models used, the type and size of microplastics, and exposure durations, complicate the generalizability of the results. This variability makes it difficult to compare results and reach general conclusions about the effects of microplastics on female reproductive health [6, 26, 43].

The results identified are critical for understanding the potential risks of microplastic exposure on reproductive health. Understanding the effects of microplastics on the female reproductive system, in particular, emerges as a vital necessity for developing environmental protection policies and efforts to safeguard human health. In this context, future research needs to address the shortcomings of existing studies to obtain more reliable and comprehensive results.

Additionally, many of the studies included in the review have methodological shortcomings, such as limited data sets and inadequate control groups. This situation restricts the full understanding of the potential effects of microplastics. Therefore, new standardized and well-designed studies are needed to more clearly reveal the effects of microplastics on reproductive health.

## Conclusion

This systematic review highlights experimental studies demonstrating the negative effects of microplastics on female reproductive health. The current results suggest that microplastic exposure may have significantly adverse effects on ovarian function, fertility, hormone levels, and embryo development. However, methodological differences and limited data among the studies make it difficult to draw definitive conclusions about the general validity of these effects. To obtain more conclusive results, there is a need for standardized and well-designed experimental studies in the future. These results underscore the potential risks of microplastic exposure on reproductive health and emphasize the need for further research in this area.

## Data Availability

This article is a systematic review. No new datasets were generated and no surveys or scales were utilized in this study. All details of the included research are presented in the relevant tables and references within the manuscript. The steps of the review process are clearly outlined in the PRISMA flow diagram. Therefore, all data analyzed in this review can be accessed through the article and its accompanying references.
